# Contribution of Calpain and Caspases to Cell Death in Cultured Monkey RPE Cells

**DOI:** 10.1167/iovs.17-22325

**Published:** 2017-10

**Authors:** Emi Nakajima, Katherine B. Hammond, Masayuki Hirata, Thomas R. Shearer, Mitsuyoshi Azuma

**Affiliations:** 1 Senju Laboratory of Ocular Sciences, Senju Pharmaceutical Corporation Limited, Portland, Oregon, United States; 2 Department of Integrative Biosciences, Oregon Health & Science University, Portland, Oregon, United States

**Keywords:** calpain, non-human primate, AMD, A2E, hypoxia

## Abstract

**Purpose:**

AMD is the leading cause of human vision loss after 65 years of age. Several mechanisms have been proposed: (1) age-related failure of the choroidal vasculature leads to loss of RPE; (2) RPE dysfunctions due to accumulation of phagocytized, but unreleased A2E (*N*-retinylidene-*N*-retinylethanolamine); (3) zinc deficiency activation of calpain and caspase proteases, leading to cell death. The purpose of the present study is to compare activation of calpain and caspase in monkey RPE cells cultured under hypoxia or with A2E.

**Methods:**

Monkey primary RPE cells were cultured under hypoxic conditions in a Gaspak pouch or cultured with synthetic A2E. Immunoblotting was used to detect activation of calpain and caspase. Calpain inhibitor, SNJ-1945, and pan-caspase inhibitor, z-VAD-fmk, were used to confirm activation of the proteases.

**Results:**

(1) Hypoxia and A2E each decreased viability of RPE cells in a time-dependent manner. (2) Incubation under hypoxia alone induced activation of calpain, but not caspases. SNJ-1945 inhibited calpain activation, but z-VAD-fmk did not. (3) Incubation with A2E alone induced activation of calpain, caspase-9, and caspase-3. SNJ-1945 inhibited calpain activation. z-VAD-fmk inhibited caspase activation, suggesting no interaction between calpain and caspases.

**Conclusions:**

Hypoxia activated the calpain pathway, while A2E activated both calpain and caspase pathways in monkey RPE cells. Such knowledge may be utilized in the treatment of AMD if inhibitor drugs against calpain and/or caspase are used to prevent RPE dysfunction caused by hypoxia or A2E.

AMD is the leading cause of human vision loss after age 65. RPE and photoreceptor (PR) cells degenerate in AMD, leading to progressive loss of central vision.^1^ The RPE is a single layer of cells between the neural retina and the choriocapillaris. Loss of RPE must impact some of the functions of normal healthy RPE, which include (1) nutrient transport to photoreceptors, (2) transport and storage of retinoids, (3) stabilization of the ion composition of the subretinal space, (4) phagocytosis and degradation of spent outer segments, (5) protection against light and free radicals, (6) isomerization of all-*trans*-retinal to 11-*cis*-retinal, and (7) secretion of essential growth factors.^2^ Risk factors for AMD are aging, hereditary, smoking, obesity, hypertension, and hypercholesterolemia.^3^ Since long-term, effective treatments are not currently available, the number of patients severely disabled by AMD is expected to increase in the next decades due to population aging.

Along with loss of RPE and PR in AMD, the stages of AMD vary according to number and size of insoluble yellow extracellular drusen deposits accumulating between the basal lamina of the RPE and the inner collagenous layer of Bruch's membrane.^4–6^ Early dry AMD shows small drusen that may not affect vision. Intermediate dry AMD is characterized by geographic atrophy, sharply delineated areas of degenerated RPE cells, and visible choroidal vessels.^7^ Late advanced AMD can be dry or wet; the latter is due to choroidal neovascularization (CNV). CNVs are newly formed immature blood vessels growing from the choroid through Bruch's membrane into the outer retina. Among these stages of AMD, several mechanisms have been proposed.

One mechanism suggested is that age-related failure of the choroid vasculature leads to loss of the RPE. Retinal blood flow is disturbed in both dry and wet AMD.^8^ Reduction in choroid perfusion has been positively correlated with progression of AMD.^3^ In later-stage wet AMD, the CNV vessels leak fluid below or into the retina. Compromised blood flow may be caused by accumulation deposits in the RPE, inflammation, and/or neovascularization. Retina is the most metabolically active tissue in the body, and it is highly sensitive to reduced oxygen.^3^ These observations suggest that poor blood and resultant hypoxia may be causative of AMD.

RPE dysfunction is due to A2E (*N*-retinylidene-*N*-retinylethanolamine). Among more than 25 components, A2E is the major fluorophore identified in lipofuscin from aged human eyes, and it has been extensively studied.^9,10^ A2E is a by-product of the visual cycle and is formed from two molecules of all-*trans*-retinal and ethanolamine. A2E accumulates in the RPE and is a hallmark of aging. The accumulation of A2E has been implicated in retinal degeneration, including AMD.^10^ A2E undergoes photo-oxidation and causes membrane permeation.^11^ The amount of lipofuscin and drusen also predicts the progression and severity of AMD. Thus, another factor in AMD development may be the accumulation of phagocytized, but unreleased toxic A2E.

Activation of calpain and caspase proteases leads to cell death in AMD. An AMD model using zinc deficiency found that mitochondrial caspase-3/-7 activation followed by calpain activation was involved in retinal cell death.^12^ Decreased zinc levels in the macula were reported in patients with AMD.^13^ The zinc content of human RPE decreases with age and in macular degeneration, and superoxide dismutase activity increases. These data suggest that activation of caspases and calpain may be responsible for proteolytic damage in the RPE of AMD patients.

To our knowledge, the relative importance of the various components presented above in the causation of AMD has not been well studied. The present study compares the activation of calpain and caspase pathways in monkey RPE cells cultured separately under hypoxic or A2E conditions. Monkey eyes were utilized because they are similar to human eyes but can be obtained more quickly after death, thus minimizing postmortem metabolic changes.

## Materials and Methods

### Experimental Animals

Eye cups from 21 rhesus macaques (*Macaca mulatta*, 1–12 years of age) were obtained at necropsy from the Oregon National Primate Research Center (Beaverton, OR, USA) from experiments unrelated to the present studies. Experimental animals were handled in accordance with the ARVO Statement for the Use of Animals in Ophthalmic and Vision Research and with the Guiding Principles in the Care and Use of Animals (DHEW Publication, NIH 80-23). The average time between death and dissection was less than 1 hour.

### Monkey Primary RPE Cell Culture

The culture method for monkey RPE cells was taken from our previous study with monkey RPE cells.^12^ Briefly, fresh eye cups were quartered, all retinal layers except RPE were removed, and the remaining RPE/choroid samples were incubated in 20 mg/mL dispase for 1 hour (MilliporeSigma, Temecula, CA, USA). RPE cells were collected by pipetting from a pair of eye cups, washed with 250 U penicillin plus 250 μg streptomycin/mL solution, and plated on a six-well plates (Greiner Bio-One, Monroe, NC, USA) with Dulbecco's modified Eagle's medium (DMEM; Thermo Fisher Scientific, Waltham, MA, USA) supplemented with 15% fetal bovine serum (FBS; PAA Laboratories, Inc., Dartmouth, MA, USA), 50 U penicillin/mL, and 50 μg streptomycin/mL at 37°C in humidified 95% air/5% CO_2_. Cells were trypsinized for passage. The cells from passage three were plated at 10^4^ cells/cm^2^ onto six-well or 96-well plates and grown for 5 days before they were used for experiments. The RPE cells reached 100% confluence on day 6.^12^ RPE cells were identified by staining for RPE marker, zonula occludens (ZO-1) (Supplementary Fig. S1). Cell phagocytic ability was confirmed by FITC-labeled bead uptake (Supplementary Fig. S2).

### Hypoxia and A2E Treatment

RPE cells were subjected to hypoxia in a gas-generating pouch system with indicator (GasPack EZ Gas Generating Pouches; Becton, Dickinson and Company, Franklin Lakes, NJ, USA) to reduce oxygen levels to ≤1% (according to the manufacturer). Hypoxic condition was confirmed with the anaerobic indicator saturated with a methylene blue solution on each sachet. This solution turns from blue to colorless in the absence of oxygen (according to the manufacturer). RPE cells were also cultured with 25 μM of A2E (Senju Pharmaceutical Co., Ltd.) in DMEM with 1% FBS and in DMEM with 0.5 mM glucose and B-27. Low glucose was present in our hypoxic group because reduced glucose has been a contributing factor in many previous ischemic injuries.^14,15^ B-27 was used to support cells after FBS was removed because it contains vitamins, essential fatty acids, hormones, and antioxidants suitable for hypoxic conditions with serum-free medium.^16^ Cells were reoxygenated in DMEM with 5.5 mM glucose (physiological glucose levels) and B-27. When used, calpain inhibitor SNJ-1945 (half maximal inhibitory concentration [IC50] for calpain 1, 0.062 μM; for calpain 2, 0.045 μM)^17^ at 10 or 100 μM, or pan-caspase inhibitor z-VAD (IC50 for caspases in yeast is 1–10.6 μM)^18^ at 100 μM (BioVision, Inc., Milpitas, CA, USA) were added 1 hour before hypoxia or A2E addition. We used a high concentration of each inhibitor because we wanted to ensure maximal inhibition of calpains and caspases, respectively (SNJ-1945 at 1 μM did not inhibit sufficiently, data not shown). Images were taken with a microscope and digitalized with a digital camera (Axiovert 200 microscope and AxioCam MRm camera; Carl Zeiss Vision Gmbh, Hallbergmoos, Germany). Images were compiled using ImageJ 1.42 (http://imagej.nih.gov/ij/; provided in the public domain by National Institutes of Health, Bethesda, MD, USA) and Adobe Photoshop (Adobe Systems Inc., San Jose, CA, USA).

### Quantitative Analysis of Cell Areas

To quantify the inhibitory effect of SNJ-1945 on the area occupied by cells under hypoxia, passage 3 cells were plated at 10^4^ cells/cm^2^ onto 48-well plates. Individual images were taken with the Axiovert 200 microscope and digitized with an AxioCam MRm camera (Carl Zeiss Vision Gmbh). Twenty images covering approximately 50% of the total well were stitched together, and the area occupied by cells was quantified using ImageJ software.

### Protein Extraction and Immunoblotting

Total cellular proteins were extracted by sonication in buffer containing 20 mM Tris (pH 7.5), 5 mM EGTA, 5 mM EDTA, and 2 mM dithioerythritol. Protein concentrations were measured by the BCA assay (Thermo Fisher Scientific) using BSA for standards. For immunoblotting, equal amounts of protein were loaded into each lane and run on 4-12% NuPAGE gels with MES or MOPS buffer (Thermo Fisher Scientific) and then electrotransferred to polyvinylidene difluoride membrane at 100 V for 1 hour. The membranes were probed with primary antibodies to calpain 1 (Thermo Fisher Scientific); calpain 2, β-actin, caspase-9 (MilliporeSigma); α-spectrin, caspase-7 (Enzo Life Sciences, Inc., Farmingdale, NY, USA); cytokeratin-18, caspase-3, caspase-8, pituitary adenylate cyclase-activating polypeptide (PACAP; Santa Cruz Biotechnology, Dallas, TX, USA); C/EBP homologous protein (CHOP), binding immunoglobulin protein (BiP), calnexin (Cell Signaling Technology, Inc., Danvers, MA, USA); and caspase-12 (Abcam, Inc., Cambridge, MA, USA). Binding of secondary antibodies, conjugated to alkaline phosphatase or to horseradish peroxidase enzyme, was visualized with 5-bromo-4-chloro-3-indolyl-phosphate/nitroblue tetrazolium (BCIP/NBT; Bio-Rad Laboratories, Hercules, CA, USA) or by chemiluminescence (Amersham ECL Plus; GF Healthcare Biosciences, Piscataway, NJ, USA). Band intensities were measured with ImageJ 1.60 software. To compensate for variability of staining between membranes, the densities of the bands were normalized to the density of β-actin. At least three independent experiments from different cultures were conducted for all studies in this report. All data shown are means ± standard error. Statistical analyses of band densities were performed by Dunnett's *t*-test (JMP 12.01 statistical software; SAS Institute Inc., Cary, NC, USA). *P* < 0.05 was considered statistically significant.

## Results

### Hypoxia/Reoxygenation Causes Activation of Calpains

**Figure 1:**
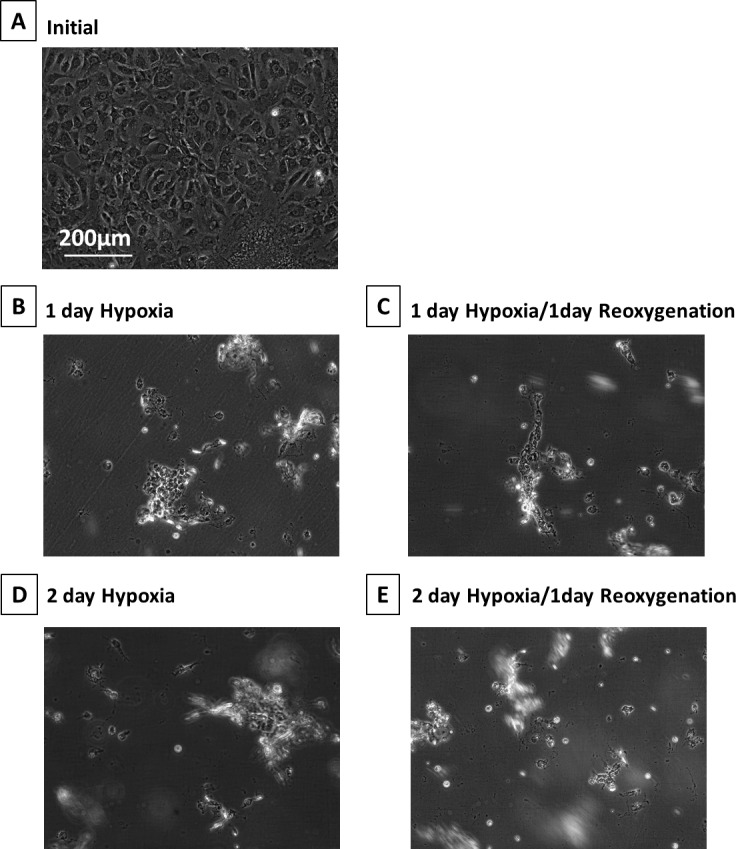
Phase-contrast micrographs of monkey RPE cells under hypoxia. (A) initial, (B) 1-day hypoxia, (C) 1-day hypoxia/1-day reoxygenation, (D) 2-day hypoxia, and (E) 2-day hypoxia/1-day reoxygenation. These images were chosen from the most representative experiment in Figure 2 (n = 3).

When cultured under hypoxia, monkey RPE cells showed severe morphologic damage starting from day 1 (Figs. 1A and B). Hypoxia/reoxygenation further damaged RPE cells in a time-dependent manner (Figs. 1A–E), and most of the cells were eliminated at day 2 of hypoxia (Fig. 1D).

**Figure 2:**
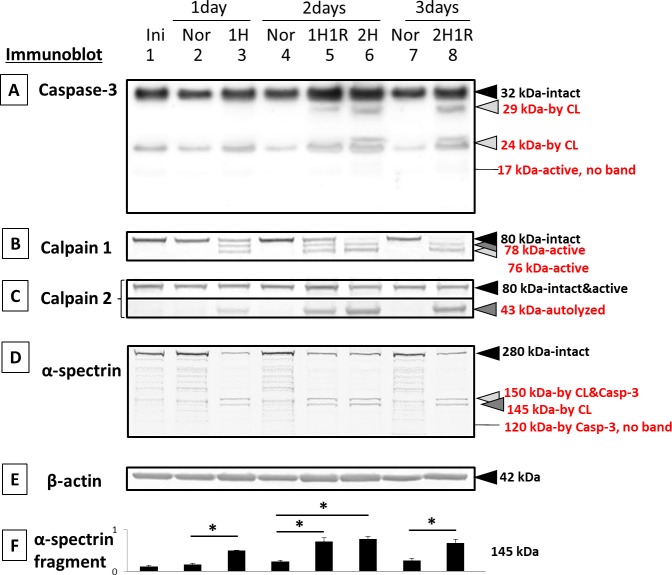
Immunoblots of caspases, calpains, and their substrates in RPE cells cultured under hypoxia: (lane 1) initial, (lane 2) 1-day normal, (lane 3) 1-day hypoxia, (lane 4) 2-day normal, (lane 5) 1-day hypoxia/1-day reoxygenation, (lane 6) 2-day hypoxia, (lane 7) 3-day normal, and (lane 8) 2-day hypoxia/1-day reoxygenation. (A) caspase-3, (B) calpain 1, (C) calpain 2, (D) α-spectrin, a substrate for both capase-3 and calpain, and (E) β-actin (nonsubstrate gel-loading control). The bar graphs show the densities of bands for (F) α-spectrin fragments at 145-kDa (calpain-specific) normalized to β-actin and expressed as means ± SEM (n = 3). *P < 0.05 relative to the corresponding normal group (Dunnett's t-test).

Hypoxia/reoxygenation caused proteolysis of pro-caspase-3 to inactive 29- and 24-kDa fragments in a time-dependent manner (Fig. 2A, lanes 5, 6, and 8). These caspase fragments are produced by calpain and are an indicator of calpain activity.^12^ The active caspase-3 fragment, at 17 kDa,^19^ was not detected (Fig. 2A).

Another indicator of calpain activation during hypoxia/reoxygenation was the detection of the active autolytic fragments of calpain 1 at 78 and 76 kDa (Fig. 2B, lanes 3, 5, 6, and 8).^20^ These active fragments were produced in a time-dependent manner. After 2 days of hypoxia, the intact band at 80 kDa was completely eliminated (Fig. 2B, lanes 6 and 8). However, because the active, *N*-terminal truncated form of calpain 2 migrates to nearly the same position as the intact 80-kDa calpain 2 on SDS-PAGE,^21^ the 80-kDa band in lanes 3, 5, 6, and 8 probably contained both intact and autolyzed calpain 2. In support of this, autolyzed calpain 2 at 43 kDa increased in a time-dependent manner (Fig. 2C). Note that calpains 1 and 2 autolytic fragments appeared on day 1 of hypoxia before the calpain-dependent caspase-3 fragments appeared (Figs. 2A–C, lane 3).

Hypoxia treatment also caused a loss of the intact α-spectrin band at 280 kDa (Fig. 2D, lanes 3, 5, 6, and 8). α-Spectrin substrate is hydrolyzed by calpains.^22^ This hydrolysis led to a significant accumulation of the calpain-specific 145-kDa fragment at 1 day after hypoxia, 1 day of hypoxia plus 1 day of reoxygenation, 2 days of hypoxia, and at 2 days of hypoxia plus 1 day of reoxygenation (Fig. 2D and F, dark gray arrowhead). β-actin was used as an internal loading control (Fig. 2E).

The data above using different substrates showed that hypoxia/reoxygenation caused time-dependent activation of calpains, but not caspases, in cultured monkey RPE cells (Fig. 2). From these data, the time point at 1 day hypoxia/1 day reoxygenation was chosen for further testing of protease inhibitors.

### Calpain, but Not Caspase Inhibitors Protect RPE Cells Under Hypoxia

**Figure 3:**
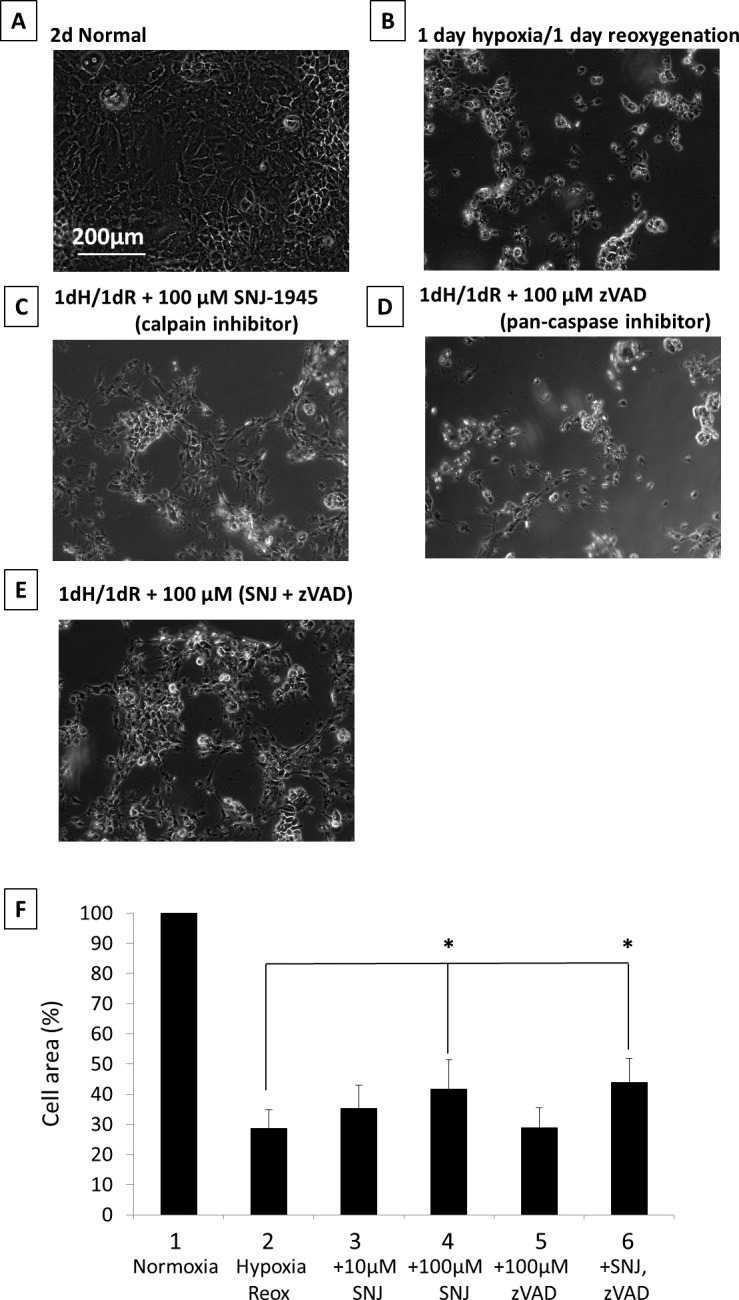
Phase-contrast micrographs of RPE cells cultured under hypoxia with inhibitors. (A) 2-day normal, (B) 1-day hypoxia/1-day reoxygenation, (C) 1-day hypoxia/1-day reoxygenation + 100 μM SNJ-1945 (calpain inhibitor), (D) 1-day hypoxia/1-day reoxygenation + 100 μM z-VAD (pan-caspase inhibitor), and (E) 1-day hypoxia/1-day reoxygenation + 100 μM SNJ-1945 + 100 μM z-VAD. These images were chosen from the most representative experiment in Figure 4 (n = 5). (F) The bar graph showing the percentage of total area occupied by attached cells in each group compared to normal cells cultured for 2 days. Data are % ± SEM (n = 5 sets, each set was an average of 20 images). *P < 0.05 relative to the hypoxia/reoxygenation group (Dunnett's t-test).

Monkey RPE cell damage under hypoxia was ameliorated by calpain inhibitor SNJ-1945 but not by pan-caspase inhibitor z-VAD (Fig. 3A–E). These results with RPE cells were similar to results with mixed cultures of retinal cells.^20^ Adding both SNJ-1945 and z-VAD together to RPE cells did not provide further protection against hypoxic damage (Fig. 3, lanes 3, 4, and 6). These visual results were quantified by measuring the cell area remaining after addition of inhibitors, and they confirmed that treatment with 100 μM SNJ-1945 resulted in the same protection as simultaneous treatment with 100 μM SNJ-1945 and 100 μM z-VAD (Fig. 3, lane 6). This suggested that neither calpain nor caspase-3 were upstream of each other or were needed for proteolytic activation of each other. Caspases-8 and -12 were not activated (data not shown), suggesting no Fas-associated protein via death domain (FADD)–inducing signal and no involvement of endoplasmic reticulum (ER)-specific proteases, respectively.

**Figure 4:**
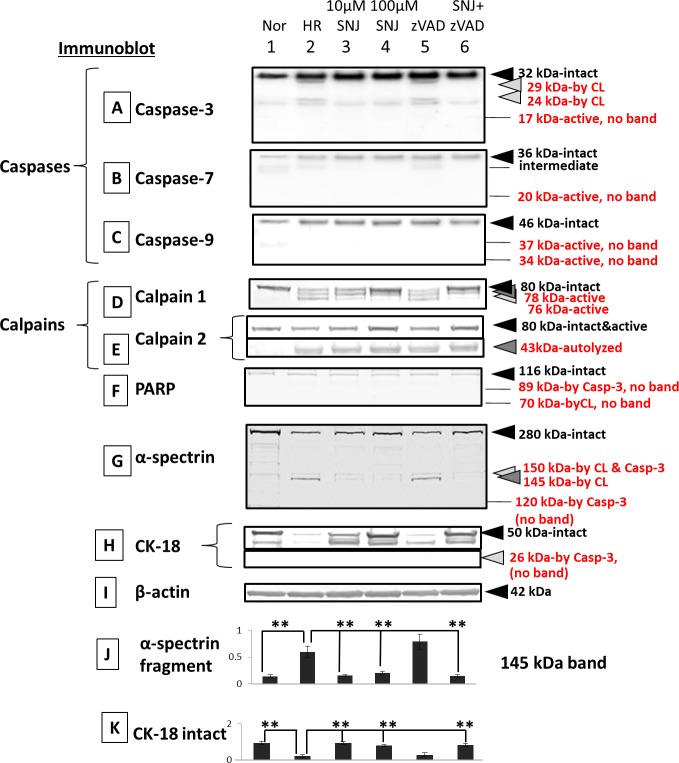
Immunoblots of caspases, calpains, and their substrates in RPE cells cultured under hypoxia with inhibitors. (Lane 1) normal, (lane 2) 1-day hypoxia/1-day reoxygenation, (lane 3) 1-day hypoxia/1-day reoxygenation + 10 μM SNJ-1945, (lane 4) 1-day hypoxia/1-day reoxygenation + 100 μM SNJ-1945, (lane 5) 1-day hypoxia/1-day reoxygenation + 100 μM z-VAD, and (lane 6) 1-day hypoxia/1-day reoxygenation + 100 μM SNJ-1945 + 100 μM z-VAD. (A) Caspase-3, (B) caspase-7, (C) caspase-9, (D) calpain 1, (E) calpain 2, (F) PARP, (G) α-spectrin, (H) RPE cell marker cytokeratin-18, and (I) β-actin. The bar graphs show the densities of bands for the (J) α-spectrin 145-kDa fragment and the (K) intact band of CK-18 normalized to β-actin. The data are expressed as means ± SEM (n = 5). *P < 0.05, all relative to the 2-day hypoxia/reoxygenation group (Dunnett's t-test).

Immunoblots showed that only calpains were activated, but caspases were not activated by hypoxia (Fig. 4A–E, lanes 1 and 2). An indicator of calpain 1 activation, at 78 and 76 kDa, was detected under hypoxia/reoxygenation, and it was rescued by SNJ-1945 in a dose-dependent manner. However, the intact band of calpain 2 at 80 kDa decreased and was rescued by SNJ-1945 but not by z-VAD under hypoxia/reoxygenation. Adding SNJ-1945 rescued calpain 2 intact band degradation. However, the calpain 2 autolyzed band at 43 kDa is still present when SNJ-1945 was added. SNJ-1945 inhibited calpain 2 activation, but it was still autodegraded. We speculate accumulated calpain 2 43-kDa band under hypoxia/reoxygenation is further degraded, so it seems that 43-kDa band intensity doesn't change by adding SNJ-1945. Similar results were observed in the monkey RPE cell culture treated with TPEN.^12^

No caspase-specific fragments from PARP, α-spectrin, or from cytokeratin-18 were produced under hypoxia (Fig. 4F–H, lanes 1 and 2).

In contrast, immunoblots showed that calpains were activated by hypoxia (Fig. 4D and E, lanes 1 and 2). Only calpain-specific fragments from α-spectrin (Fig. 4G, lanes 1 and 2) were observed. Loss of the intact band of cytokeratin-18 was observed, but this was not by caspase-3 (Fig. 4H, lanes 1 and 2). Caspase-3 was degraded by calpain to the known calpain-dependent cleavage products at 24 and 29 kDa. These fragments were not further cleaved (Fig. 4A, lanes 1 and 2). SNJ-1945 inhibited calpain activation (Fig. 4D and E, lanes 2–4 and 6). SNJ-1945 inhibited proteolysis of calpain substrates α-spectrin (Fig. 4G, lanes 2–4 and 6) and cytokeratin-18 (Fig. 4H, lanes 2–4 and 6). In the present study, calpain-dependent cleavage band from PARP at 70 kDa did not appear (Fig. 4F, lanes 2–4 and 6). z-VAD did not inhibit any proteolysis occurring during hypoxia (Fig. 4A–H, lanes 2, 5, and 6). Densitometric analysis of the immunoblots for the calpain-specific spectrin breakdown product at 145 kDa and for the loss of the intact cytokeratin-18 band confirmed significant inhibition of proteolysis by SNJ-1945, but not by z-VAD (Fig. 4J and K). These data further confirmed that only calpain caused hypoxia-induced RPE cell damage. Again, these data were similar to results in mixed monkey retinal cells cultured under hypoxia.^20^

### Activation of Calpains and Caspase-3 During A2E Treatment

**Figure 5:**
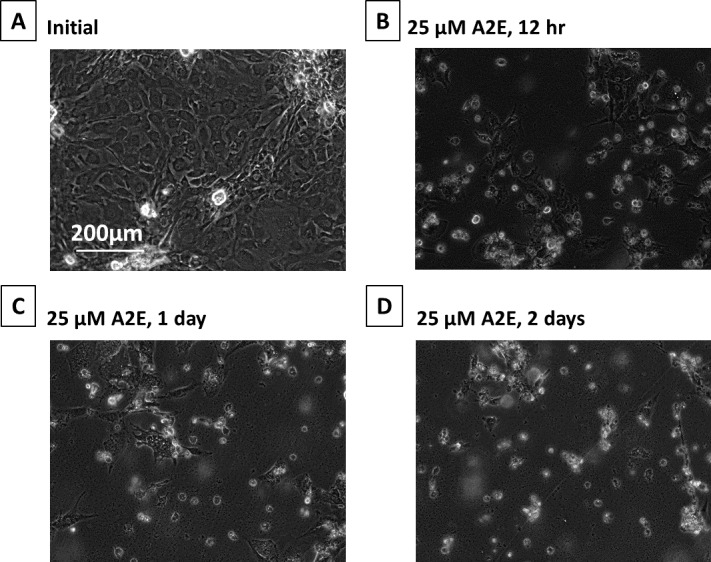
Phase-contrast micrographs of RPE cells cultured with A2E. (A) Initial, (B) plus 25 μM A2E at 12 hours, (C) 25 μM A2E at day 1, and (D) 25 μM A2E at day 2. These images were chosen from the most representative experiment in Figure 6 (n = 3).

A2E treatment of monkey RPE cells caused severe morphologic damage starting at 12 hours (Fig. 5A versus B). Longer treatment with A2E further damaged RPE cells so that only debris resembling rounded cells remained by day 2 (Fig. 5C and D).

**Figure 6:**
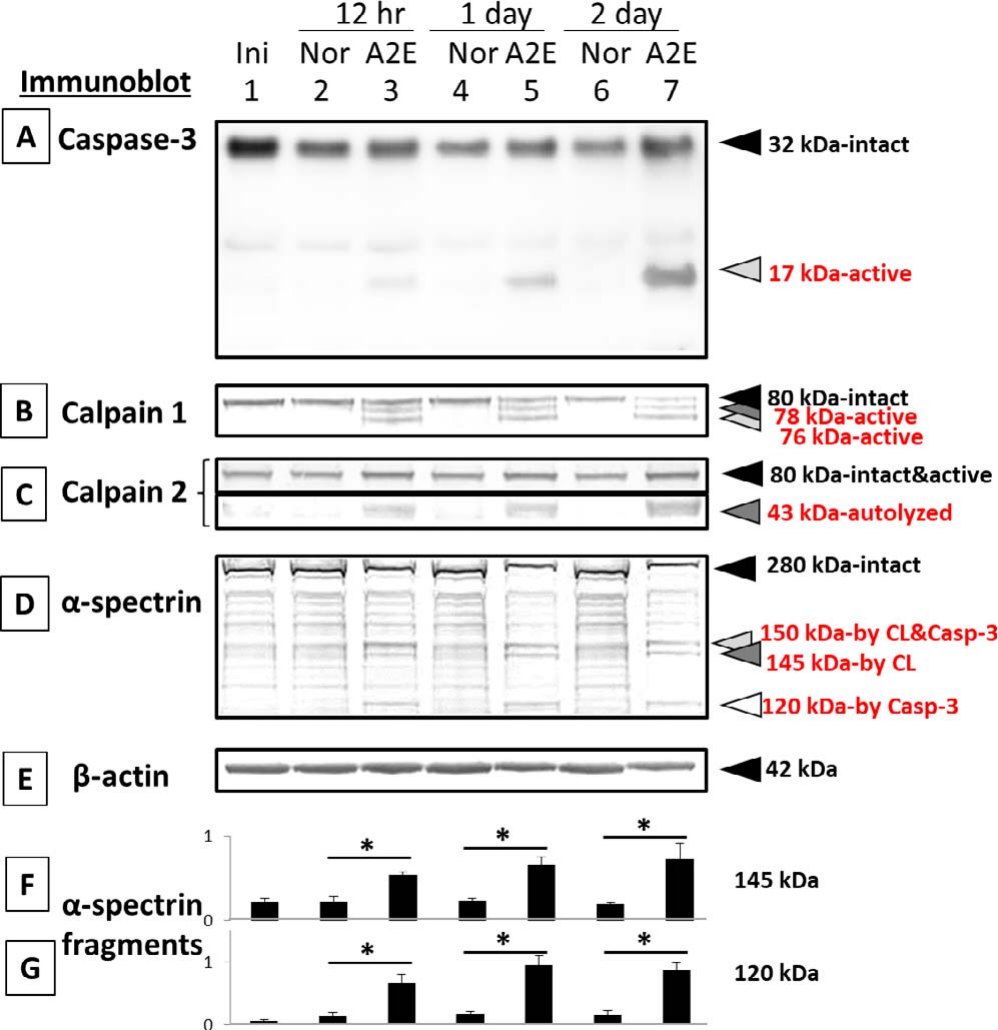
Immunoblots of caspases, calpains, and their substrates in RPE cells cultured with A2E. (Lane 1) initial, (lane 2) 12-hour normal, (lane 3) 12-hour 25 μM A2E, (lane 4) 1-day normal, (lane 5) 1-day 25 μM A2E, (lane 6) 2-day normal, and (lane 7) 2-day 25 μM A2E. (A) Caspase-3, (B) calpain 1, (C) calpain 2, (D) α-spectrin, and (E) β-actin. Bar graphs show the densities of α-spectrin fragments at (F) 145 kDa (calpain-specific) and at (G) 120-kDa (caspase-3-specific) normalized to β-actin. Data are expressed as means ± SEM (n = 3). *P < 0.05 relative to the corresponding normal group (Dunnett's t-test).

In the A2E-treated cells, endogenous pro-caspase-3 at 32 kDa was proteolyzed to an active 17-kDa fragment (Fig. 6A). The smaller, active caspase-3 fragment at 12 kDa, known to form an active capase-3 heterodimer with the 17-kDa fragment, was not detected. Also, inactive caspase-3 fragments at 29 and 24 kDa caused by calpain were not present (Fig. 6A, lanes 2–7).

Autolysis of calpains is associated with their activation.^21^ The intact 80-kDa catalytic subunit of low calcium-requiring calpain 1 decreased after A2E treatment, and active autolytic fragments at 78 and 76 kDa appeared (Fig. 6B, lanes 3, 5, and 7). Similarly, an autolyzed fragment of calpain 2 at 43 kDa increased in a time-dependent manner (Fig. 6C, lanes 3, 5, and 7).

A2E treatment also caused a loss of intact 280-kDa α-spectrin (Fig. 6D). This substrate is hydrolyzed by both caspase-3 and calpains (Fig. 6D, lanes 3, 5, and 7). This led to a statistically significant accumulation of a calpain-specific 145-kDa fragments at 12 hours, 1 day, and 2 days (dark gray arrowhead, Fig. 6D and F) and a caspase-3 specific 120-kDa fragment at 12 hours and days 1 and 2 (open arrowhead, Fig. 6D and G). β-actin was used as an internal loading control (Fig. 6E). These results showed that 25 μM A2E causes a time-dependent activation of both caspase-3 and calpain in cultured monkey RPE cells. The 1-day time point was chosen for further testing of enzyme inhibitors as shown below.

### Calpain and Caspase Inhibitors Partially Protected RPE Cells From A2E damage—1 Day

**Figure 7:**
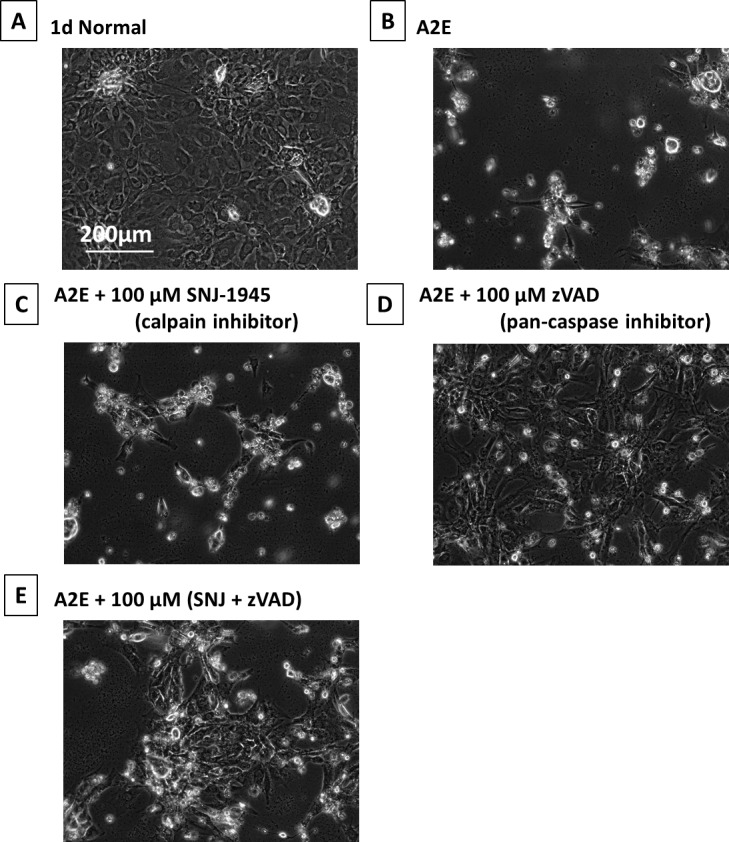
Phase-contrast micrographs of RPE cells cultured with A2E and inhibitors. (A) 1-day normal, (B) plus 25 μM A2E at 1 day, (C) 25 μM A2E + 100 μM SNJ-1945, (D) 25 μM A2E + 100 μM z-VAD, and (E) 25 μM A2E + 100 μM SNJ-1945 + 100 μM z-VAD. These images were chosen from the most representative experiment in Figure 8 (n = 5).

Unexpectedly, A2E still caused morphologic damage to RPE treated with calpain inhibitor SNJ-1945 and/or pan-caspase inhibitor z-VAD (Fig. 7A–E). Shrunken cells were partially lost with SNJ-1945 (Fig. 7C) but not lost with z-VAD (Fig. 7D). Adding SNJ-1945 and z-VAD together did not further protect the cells (Fig. 7E). We did not measure the cell survival area in the A2E experiment for the minimum of animal usage, but we expect the results to be similar to the images under hypoxia (Fig. 3F).

**Figure 8:**
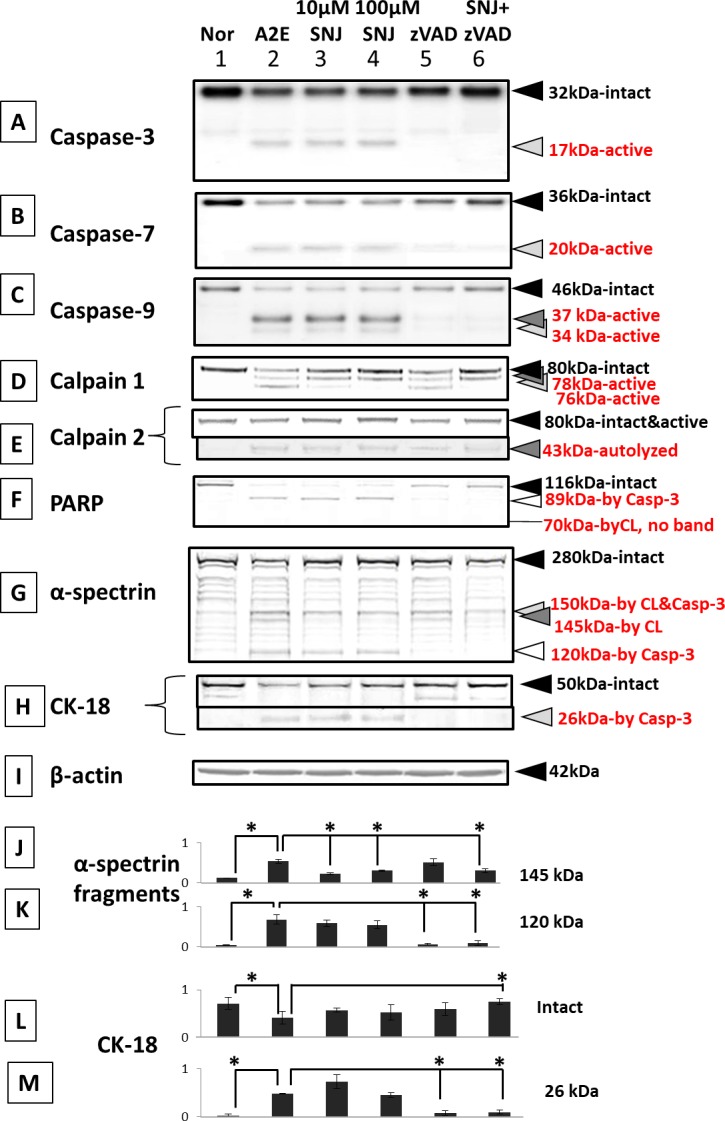
Immunoblots for caspases, calpains, and their substrates in RPE cells cultured with A2E and inhibitors. (Lane 1) normal, (lane 2) 1-day 25 μM A2E, (lane 3) 25 μM A2E + 10 μM SNJ-1945, (lane 4) 25 μM A2E + 100 μM SNJ-1945, (lane 5) 25 μM A2E + 100 μM z-VAD, and (lane 6) 25 μM A2E + 100 μM SNJ-1945 + 100 μM z-VAD. (A) Caspase-3, (B) caspase-7, (C) caspase-9, (D) calpain 1, (E) calpain 2, (F) PARP, (G) α-spectrin, (H) intact cytokeratin-18 (CK-18) and CK-18 fragment at 26 kDa (caspase-3-specific), and (I) β-actin. The bar graphs show the densities of the bands for the (J) α-spectrin 145-kDa fragment, (K) α-spectrin 120-kDa fragment, (L) CK-18 intact band, and (M) the 26-kDa CK-18 fragment, all normalized to β-actin. Data are expressed as means ± SEM (n = 5). *P < 0.05 relative to A2E (Dunnett's t-test).

Calpain inhibitor SNJ-1945 prevented only calpain activation (Fig. 8D, lanes 2–4 and 6). Here again the calpain 2 autolyzed band at 43 kDa is still present when SNJ-1945 was added. The 43-kDa band is further degraded, so it seems that when the calpain inhibitor is present, this further degradation is inhibited (Fig. 8E).

Caspase inhibitor z-VAD partially inhibited activation of calpain and mitochondrial caspases-3, -7, and -9 (Fig. 8A–E, lanes 2, 5, and 6). These data suggest that mitochondrial caspases might be upstream of calpain during A2E cell damage. Caspases-8 and -12 were also not activated (data not shown).

SNJ-1945 inhibited formation of the calpain-specific spectrin breakdown product (SBDP) at 145 kDa, but did not inhibit caspase-specific 120 kDa (Fig. 8G, lanes 2–4 and 6). Caspase inhibitor z-VAD inhibited formation of 120-KDa SBDP, but did not inhibit formation of the calpain-specific 145-kDa SBDP (Fig. 8G, lanes 2, 5, and 6). PARP and cytokeratin-18 are caspase-3 substrates and produce hydrolysis fragments. Note that z-VAD prevented loss of these breakdown fragments (Fig. 8F and H, lanes 2, 5, and 6).

Densitometric analysis of the calpain-specific 145-kDa SBDP confirmed significant inhibition by SNJ-1945, but not by z-VAD (Fig. 8J). The analysis also showed significant inhibition of caspase-induced proteolysis (120-kDa SBDP production) by z-VAD, but not by SNJ-1945(Fig. 8K).

Densitometric analysis of intact cytokeratin-18 and its 26-kDa caspase-specific breakdown band confirmed preservation of cytokeratin-18 by SNJ-1945 only when it was paired with z-VAD (Fig. 8L). By itself, z-VAD prevented formation of the 26-kDa fragment (Fig. 8M).

## Discussion

These studies delineated mechanisms relating to the involvement and interrelationships of four factors (hypoxia, A2E, calpains, and caspases) possibly related to the basic mechanism of AMD.

### Hypoxia Alone Activates Calpain-Induced Proteolysis

**Figure 9:**
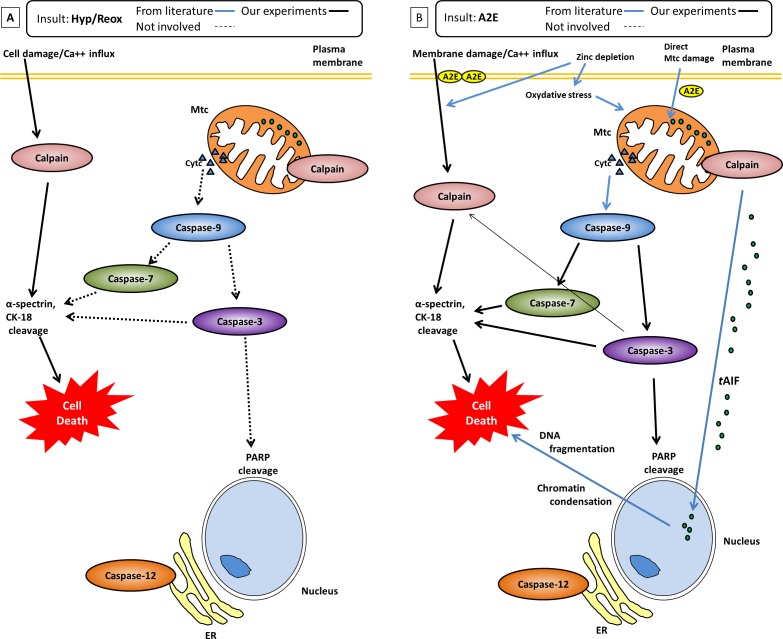
(A) Hypoxia-induced cell death in RPE cells is due only to activation of calpains. (B) Proposed pathway leading to A2E–induced cell death due to activation of caspase-3, -7, and -9, and calpains. Solid lines show the pathways confirmed in present study, blue lines show the pathways reported in the literature. Caspases-8 and -12 were not activated by either hypoxia or A2E.

Specifically, we found RPE under hypoxic conditions showed calpain activation, but not caspase activation (Fig. 3B–F). Our hypothesis (Fig. 9A) for the hypoxic damage to RPE cells is first, hypoxia causes plasma cell membrane damage, leading to increased calcium flux into the cytosol. The free calcium activates calpains.^20^ Note that, unlike most proteases, calpains are activated directly by binding of calcium to the calpain molecule itself.^23^ Second, calpains proteolyze cytoskeletal proteins α-spectrin and cytokeratin-18. These proteolytic events cause severe damage and cell death.^12,24^ Neither mitochondrial caspases, ER stress (caspase-12 activation), nor caspase-8 involvement were observed in hypoxia. Thus, calpain-induced proteolysis, not caspase activity, leads to the cell damage observed in our RPE cells cultured under hypoxia.

Hypoxia/reoxygenation conditions are relevant to the human situation because oxidative stress has been observed in aged RPE cells and implicated in the pathogenesis of AMD.^3^ Oxidative stress occurs when the level of reactive oxygen species (ROS) exceeds the detoxifying capacity of antioxidants and/or overwhelms the protective action of molecular chaperones. Excess ROS damage RPE cell membranes and weakens their ability to remove the constant stream of lipofuscin metabolites derived from the rods and cones.^3^ Accumulation of lipofuscin in lysosomes further magnifies ROS production in the RPE cells. ROS also mediate prolyl hydroxylase activity and increase hypoxia-inducible factor (HIF) expression. Increased HIF expression and stability cause overexpression of VEGF and leads to angiogenesis in the RPE.^3^

The retina responds to hypoxia by HIF-induced overproduction of VEGF (disrupting the blood–retinal barrier), production of inflammatory cytokines, increasing NO production (enhanced free radical production along with vasodilation), enhancing extracellular glutamate that results in increased calcium flux into the cytoplasm, and decreased oxygen-dependent energy production (with loss of ATPase-linked calcium channels).

A common messenger in many of these response pathways is increased cytoplasmic free calcium. In monkey retina under hypoxic conditions, mitochondrial damage is not apparent, yet calpain activation occurs through influx of calcium.^20^ This was observed in both monkey and human retinal tissues cultured under hypoxic condition,^25^ as well as in the present cultured monkey RPE cells.

### A2E Causes Caspase-3 and Calpain-Induced Proteolysis in RPE Cell Death

To our knowledge, the current study is the first to show that calpain is involved in the mechanism for A2E damage to RPE cells. This helps explain the numerous in vitro studies showing A2E toxicity.^9–11^ Further, the current study using primate cells is a better model for AMD research because A2E and lipofuscin distribution in primate retina is different from that in mice.^9,26^ Specifically, the present study showed that A2E activated calpain and caspases. Our data suggested that mitochondrial caspases might be upstream of calpain. TPEN activation of calpain was likewise shown to be a caspase-independent event.^12^ A2E- and TPEN-treated cells did show slight differences, but in both cases, cytosolic calpain and mitochondrial caspases were involved. This suggests A2E-induced signaling cascades are similar to TPEN signaling cascades.

Our hypothesis (Fig. 9B) for the culpable signaling cascade for the toxicity of A2E in RPE cells is that A2E disrupts the plasma membrane leading to increased calcium flux into the cytosol^27^ and calpain activation, and damage to the mitochondrial membrane releases cytochrome c (Cyt c) and caspase-9 into the cytosol. Formation of the apoptosome APAF-1 complex from Cyt c and pro-caspase-9 activates caspase-9. Caspase-9 activates effector caspases-3 and -7 and leads to cell death. We also hypothesize that calpain-dependent truncation of apoptosis-inducible factor (AIF) causes AIF translocation (*t*AIF) from mitochondria to nucleus, where it causes chromatin condensation,^28^ and that caspases and calpains proteolyzed cytoskeletal proteins α-spectrin and cytokeratin-18, further adding to severe cell damage and death.^12,24^

A2E showed a similar signal cascade caused cell damage, as did our previous report with the TPEN, zinc depletion model.^12^

A limitation to these studies is that they were performed in cultured non-human primate cells that likely do not react to hypoxia or A2E exactly as in the human AMD patient. Low glucose was also present in our hypoxic group in accordance with other previous ischemic studies.^14,15^ The studies do, however, provide a molecular-based, cogent hypothesis for testing calpain inhibitors in human RPE cells and possible clinical trials. This is challenging regarding human safety since calpains are found in nearly all cells. However, in addition to ophthalmic diseases, a recent review lists more than 30 diseases or conditions in which calpain inhibition has been studied.^23^ Thus, calpain inhibition is a current active area in pretranslational research.

### Summary

Data presented support that inhibition of cytosolic calpains and mitochondrial caspases in RPE may be useful in understanding the mechanisms of AMD, a disease that is accompanied by hypoxia and/or increased A2E. Our hope is that inhibitor drugs against calpain and/or caspases would be effective in ameliorating human RPE dysfunction where hypoxic conditions due to ocular vascular failure are present.

## Supplementary Material

Supplement 1
