# Supplementary material for: Contribution of Calpain and Caspases to Cell Death in Cultured Monkey RPE Cells
Source: Invest Ophthalmol Vis Sci. 2017 Oct;58(12):5412–20. doi: 10.1167/iovs.17-22325 (PMC6110128; doi:10.1167/iovs.17-22325)
Supplement: Supplement 1 [file iovs-58-12-16-s01.pdf]

Nakajima et al., Supplemental Data

Fig. S1

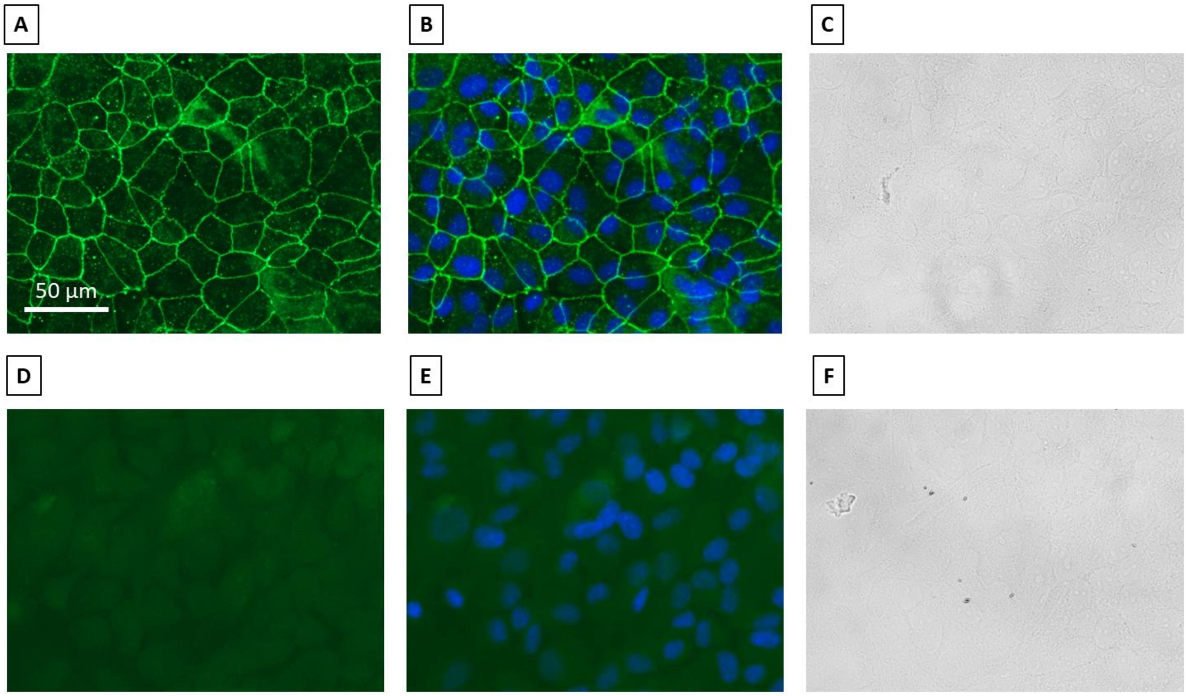

Supplemental Figure S1. Immunocytochemistry of cultured passage 3 RPE cells. (A) ZO-1 staining (green), (B) merged image with DAPI (blue), (C) corresponding phase-contrast image, (D) negative control (green), (E) merged image with DAPI (blue) and (F) corresponding phase-contrast image. These images were chosen from the most representative of 3 experiments.

Methods: Cells were grown on cell culture-treated, glass, 8-well chambers, and then were fixed for 30 min at room temperature in phosphate-buffered saline (PBS; Thermo Fisher Scientific) containing 10% formalin followed by immunocytochemistry. The cells were blocked with 10% goat IgG in PBS for 30 min at room temperature. The cells were then incubated 2 h at room temperature with primary antibody for zonula occludens (ZO-1; Cell Signaling Technology, Danvers, MA). For negative controls, the cells were incubated with normal goat IgG. After the cells were rinsed with PBS, they were incubated with Alexa-Fluor 488-labeled anti-rabbit immunogloblin (IgG; Thermo Fisher Scientific) and 1 µg nuclear stain 4',6-diamidino-2-phenylindole, dihydrochloride (DAPI; Thermo Fisher Scientific)/mL for 1 h at room temperature.

All the primary and secondary antibodies were diluted 1:200 in 5% goat IgG in PBS. The fluorescence images were photographed using an inverted microscope (Axio Observer), equipped with an AxioCam MRm camera (Carl Zeiss Vision Gmbh, Hallbergmoos, Germany). Images were complied in ImageJ 1.51n software (developed by Wayne Rasband, National Institutes of Health, Bethesda, MD).

Fig. S2

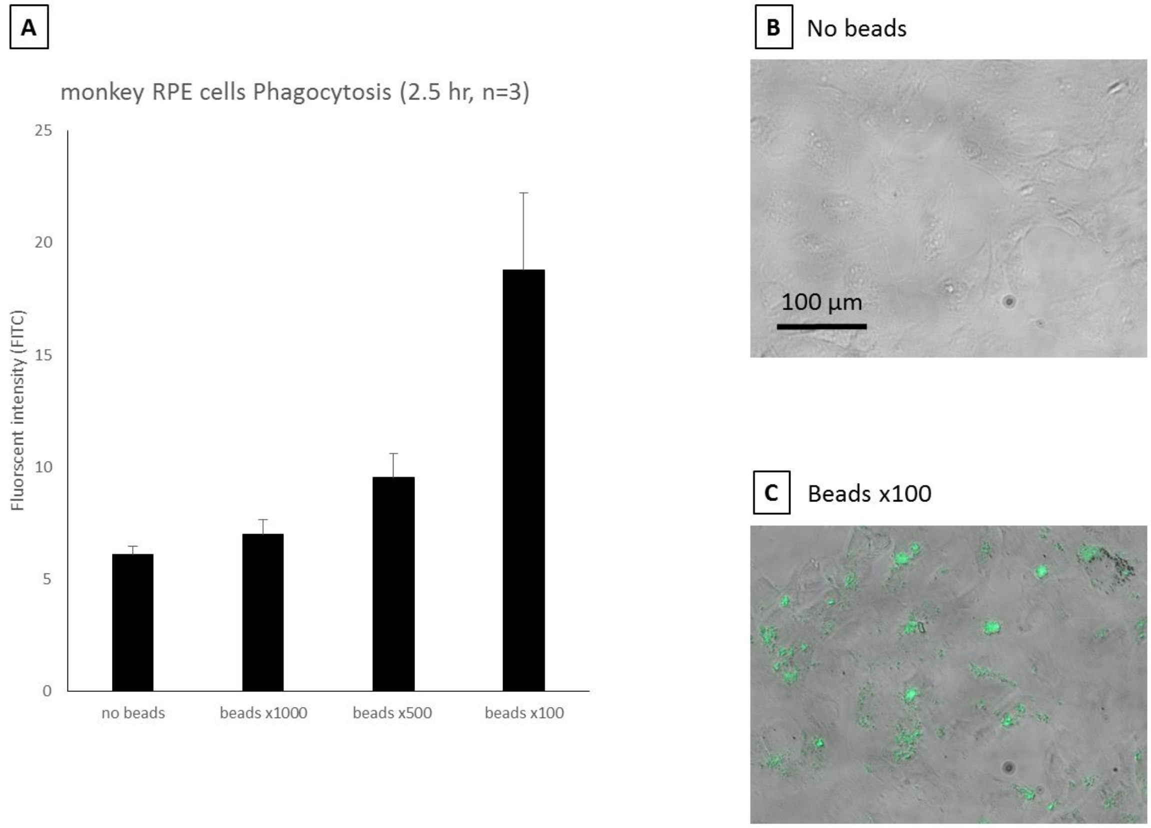

Supplemental Figure S2. **(A) Phagocytosis assay of passage 3 RPE cells. (B) negative control (no beads added) and (C) with latex beads (green). Images were merged with their phase-contrast images.**

Methods: To test the ability of RPE cells to phagocytize in vitro, cells were grown to confluence as monolayers on tissue culture-treated plastic plates, incubated for 2.5 hrs with 1 µm green fluorescent latex beads, washed with PBS three times, and the fluorescence was measured immediately. The cells were fixed for 30 min at room temperature in PBS containing 10%

formalin for imaging. The images were photographed using Axio Observer with an AxioCam MRm camera.
